# Older Adults with Weaker Muscle Strength Stand up from a Sitting Position with More Dynamic Trunk Use

**DOI:** 10.3390/s18041235

**Published:** 2018-04-17

**Authors:** Rob C. van Lummel, Jordi Evers, Martijn Niessen, Peter J. Beek, Jaap H. van Dieën

**Affiliations:** 1 McRoberts, Raamweg 43, 2596 HN The Hague, The Netherlands; J.Evers@mcroberts.nl (J.E.); M.Niessen@mcroberts.nl (M.N.); 2 Department of Human Movement Sciences, Faculty of Behavioural and Movement Sciences, Vrije Universiteit Amsterdam, Amsterdam Movement Sciences, Van der Boechorststraat 9, 1081 BT Amsterdam, The Netherlands; p.j.beek@vu.nl (P.J.B.); j.van.dieen@vu.nl (J.H.v.D.)

**Keywords:** physical function, physical performance test, chair stand, sit to stand transfer, wearables, inertial sensors, accelerometers, gyroscopes

## Abstract

The ability to stand up from a sitting position is essential for older adults to live independently. Body-fixed inertial sensors may provide an approach for quantifying the sit-to-stand (STS) in clinical settings. The aim of this study was to determine whether measurements of STS movements using body-fixed sensors yield parameters that are informative regarding changes in STS performance in older adults with reduced muscle strength. In twenty-seven healthy older adults, handgrip strength was assessed as a proxy for overall muscle strength. Subjects were asked to stand up from a chair placed at three heights. Trunk movements were measured using an inertial sensor fixed to the back. Duration, angular range, and maximum angular velocity of STS phases, as well as the vertical velocity of the extension phase, were calculated. Backwards elimination using Generalized Estimating Equations was used to determine if handgrip strength predicted the STS durations and trunk kinematics. Weaker subjects (i.e., with lower handgrip strength) were slower during the STS and showed a larger flexion angular range and a larger extension angular range. In addition, weaker subjects showed a greater maximum angular velocity, which increased with lower seat heights. Measurements with a single inertial sensor did reveal that older adults with lower handgrip strength employed a different strategy to stand up from a sitting position, involving more dynamic use of the trunk. This effect was greatest when elevating body mass. Trunk kinematic parameters were more sensitive to reduced muscle strength than durations.

## 1. Introduction

The ability to stand up from a sitting position is essential for older adults to live independently and maintain an adequate level of physical activity. Older adults with a better sit-to-stand (STS) performance compared to age-matched controls showed shorter sitting periods, longer standing periods, and a higher number of locomotion periods in daily life, which indicates a more active lifestyle [1]. In community-dwelling older adults, sedentary behavior caused in part by difficulty to stand up was associated with increased risk of sarcopenia [2] and mortality [3,4,5].

Previous research clarified the dynamics of the STS movement to better understand its dynamics. Schenkman et al. distinguished four STS phases, which include the flexion momentum phase, the momentum transfer phase, the vertical extension phase, and the stabilization phase [6]. Riley et al. found indications that the momentum transfer phase, which starts with the lift-off from the chair seat, represents the most demanding phase [7]. The STS involves a transition from an intrinsically stable three-point support to a dynamically stable two-point support [7]. Difficulty in rising from a seated position may directly increase the risk of injury since STS transfers were found to be responsible for 41% of all falls in nursing homes [8].

Muscle strength represents one of the most important factors contributing to success in rising from a chair [9] and has been identified as a factor determining the lowest chair height from which the functionally impaired elderly can rise [10]. STS transitions require the development of substantial muscle power and consequently many older adults perform such transitions close to their maximal ability [10,11]. In addition, functionally limited elderly individuals with lower quadriceps muscle strength showed a lower dynamic stability when performing the STS at their preferred speed [12]. Several studies have reported different strategies for standing up [13,14,15]. In general, functionally impaired elderly stand up with greater flexion of the upper body [16], possibly to achieve better postural stability [13]. Age-related reduction in muscle strength may determine the choice of STS strategy [17]. Using a muscle-actuated optimal control model, Bobbert et al. [18] found that optimizing the STS strategy can reduce the mechanical demands on all muscles by 45% compared to a normal STS. In particular, an STS strategy with greater trunk flexion led to reduced demands on the knee extensors. This finding is in line with the effects that are observed when subjects are instructed to increase trunk flexion [14]. Overall, the existing body of evidence suggests that older adults, especially adults with muscle weakness, may show adaptations in the manner in which they perform the STS.

In clinical practice, STS performance is usually evaluated by measuring the time it takes to perform a series of STS movements. Since the early 1990s, body-fixed inertial sensors have been used to quantify STS movements [19,20,21,22,23,24]. Single sensor instrumentation can be easily applied in an unobtrusive manner with high measurement reliability in a geriatric setting [25]. Instrumented measurements of STS performance yield several additional parameters that may provide a basis for a quantitative assessment of STS performance in a clinical practice setting [26]. Repeated STS movements with automatically detected sub-durations were proven to have stronger associations with health status, functional status, and physical activity than manually recorded STS durations, which implies greater clinical relevance outcomes of instrumented analysis than manually recorded durations [27]. Furthermore, instrumented analysis allowed for the assessment of the test’s dynamic phases, which are likely more informative than the static sitting and standing phases [27].

The aim of this study was to investigate whether measurements of STS movements using body-fixed sensors yield parameters that are informative regarding changes in the STS kinematics in older adults with reduced muscle strength by using handgrip strength (HGS) as a proxy for overall muscle strength. HGS is an easy to use method to measure muscle strength in a clinical practice setting and has frequently been associated with overall and lower extremity muscle strength [28,29,30]. It was hypothesized that weaker adults (i.e., with lower handgrip strength) would be inclined to use an STS strategy involving a more dynamic use of the trunk.

## 2. Materials and Methods

### 2.1. Participants

Twenty-seven healthy older adults, living in sheltered housing or in the community (12 females; mean age: 73.8 ± 7.9 years; mean weight: 77.1 ± 13.2 kg; mean height: 173.4 ± 7.4 cm) participated in this cross-sectional study. The protocol was approved by the ethics committee of the Department of Human Movement Sciences of the Vrije Universiteit Amsterdam (ECB 2014-3M) before it was conducted. Prior to testing, all participants provided written informed consent.

### 2.2. Instrumentation and Data Acquisition

**Figure 1:**
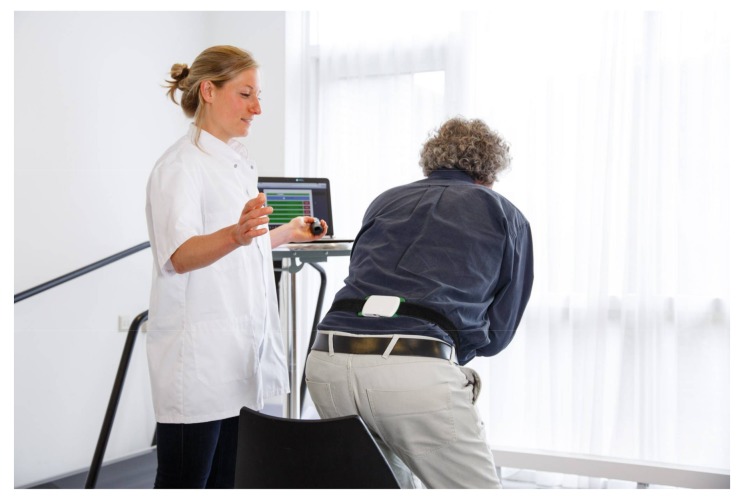
The experimenter stayed in close proximity to the patient. The protocol was implemented on a computer. With a remote control, the start and end of every STS was marked and stored with the raw data.

Trunk movements during STS were measured using a small and light (87 × 45 × 14 mm, 74 g) inertial sensor measurement system (DynaPort Hybrid, McRoberts, The Hague, The Netherlands), which was fixed with an elastic belt around the waist and placed over the spine. The experimental set-up is depicted in Figure 1.

**Figure 2:**
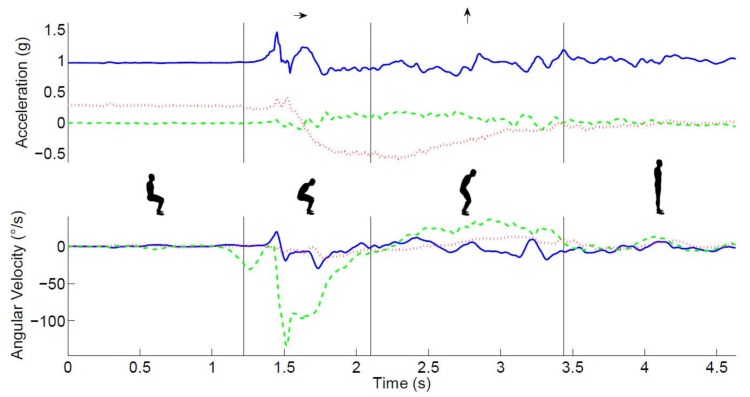
The main phases of the STS movement. The top panel shows the time series of raw acceleration (green/dashed-mediolateral, red/dotted anteroposterior, and blue/solid vertical), the lower panel shows the angular velocity (green/dashed-pitch, red/dotted-roll, and blue/solid -yaw) signals, and the insets illustrate the main phases of the STS movement, which are separated by vertical lines in both graphs.

This sensor measured acceleration and angular velocity in three directions at a rate of 100 samples/s (see Figure 2). A single sensor was used because this is more practical for clinical use than multiple sensors. A position near the center of mass was chosen to obtain an adequate reflection of the whole body movement [31]. Moreover, at this position the sensor was unobtrusive, easy to fasten, and did not hamper the participant’s movements.

### 2.3. Test Protocol

Participants were asked to stand up at their preferred speed from a height adjustable chair because effects of muscle strength are likely to be greater when standing up from lower chairs. Practice was allowed if needed. After standing up, participants were required to stand still for 10 s. Each participant performed two STS movements from three different chair heights at 100%, 90%, and 80%. The 100% chair height was defined according to the operational definition used by Schenkman [6] with the chair adjusted in such a manner that the participant’s thighs were horizontal with shanks and feet situated in a preferred orientation to stand up. The three chair heights were determined prior to performing the trials. The STS protocol was implemented on a computer, which randomly assigned the order of the height conditions. Markers set with the remote control at the start and end of each trial were stored with the raw signals to enable automatic data analysis. The three height conditions were performed with the arms folded in front of the trunk. When participants were unable to perform a certain condition, it was skipped and the next condition was offered. 

### 2.4. Signal Analysis

The measurement of three-dimensional accelerations and angular velocities of the trunk allowed for a detailed analysis of the STS movement’s different phases (see Figure 2). The acceleration and the angular velocity in the sagittal plane were used to calculate the trunk pitch angle [32]. Drift and noise were removed from the trunk pitch angle using the discrete wavelet transformation [19]. The dips in the trunk angle were used to detect a change in the trunk rotation direction. The start of the STS was defined as the end of the plateau before the first dip in the trunk pitch angle. Similarly, the end of the sit-to-stand was defined as the start of the plateau after the first dip in the trunk pitch angle. ‘True vertical acceleration’ was estimated by removing the influence of the trunk pitch angle from the vertical acceleration signal. Subsequently, the vertical velocity was derived by integrating this signal.

The following events were defined using commercially available software (MoveTest^®^, McRoberts^®^, The Hague, The Netherlands): the start of trunk movement, the end of the trunk flexion phase, and the end of the trunk rising phase [33] (see Figure 2). After automated identification of these phases, the duration, angular range, and maximum angular velocity of these phases as well as the vertical velocity of the extension phase were calculated. The mean of the two repetitions was used for statistical analysis.

### 2.5. Handgrip Strength (HGS)

HGS of both hands was measured using a digital handgrip dynamometer (Takei A5401) following a standardized protocol. Participants were standing in an upright position with their arms alongside their body. The handle was adjusted to fit their hand size. They were asked to squeeze the dynamometer as forcefully as possible.

Maximal HGS was measured twice for both hands with brief pauses between each measurement. The mean of all four measurements was used to estimate HGS. To assess the maximal HGS, at least three attempts are needed if HGS is considered to be a continuous variable while two attempts are sufficient to assess age-associated loss of muscle strength not caused by neurological or muscular diseases (dynapenia) [34].

### 2.6. Statistical Analysis

Normally distributed characteristics were presented as the mean and standard deviation (SD). Skewed (non-Gaussian) distributed continuous variables were presented as the median and interquartile range (IQR). 

#### 2.6.1. Checks for Normality

STS parameters were checked for normality using the Kolmogorov-Smirnov test. Out of a total of 24 parameters, 8 were not normally distributed. Total STS duration and the duration of the flexion phase did not meet the criteria for normality for all three seat heights. For these duration parameters, skewness and kurtosis ranged from 2.0 to 2.1 and from 3.0 to 5.9, respectively. Also non-normal distribution included the maximum angular velocity during the flexion phase at the 80% chair height (D(24) = 0.187, *p* < 0.05) and the duration of the extension phase at the 90% seat height (D(25) = 0.206, *p* < 0.05).

#### 2.6.2. Analysis

All outcome variables were analyzed using Generalized Estimating Equations (GEE) to examine whether they were associated with HGS. GEE was chosen instead of a conventional least-squares regression because it can cope with missing values and takes into account that observations within a data set are dependent. In our study, these were the different seat heights for all subjects. In addition, body length, weight, age, and gender were treated as potential confounders. The main effects were calculated for HGS and these potential confounders. Because the effect of handgrip force was of interest and an interaction with task difficulty was plausible, the interaction between handgrip strength and seat height was also included in the initial model. The first iteration of the GEE analysis included HGS, HGS*seat height, and all potential confounders. A backward elimination method was used to eliminate non-significant predictors. Seat height was always included in the model since this was the within-subject variable. All analyses were conducted in SPSS v. 21.0. 

## 3. Results

From the 27 participants, 24 completed the entire protocol. One participant was unable to stand up from the 80% chair height while two participants were unable to stand up from both the 90% and 80% chair heights. All participants (i.e., also the three participants that did not complete the entire protocol) were included in the analysis. Participant characteristics and descriptive statistics are presented in Table 1.

The results of the durations and kinematics of the three seat heights and the model effects of GEE are presented in Table 2.

### 3.1. Sit-To-Stand

There was no significant effect of seat height on STS duration (*p* = 0.097). HGS was the only significant covariate showing that weaker subjects had a longer STS duration (*p* = 0.015).

### 3.2. Flexion Phase

There was no significant effect of seat height on flexion duration (*p* = 0.071). Angular range increased with lower seat height (*p* = 0.003), weaker subjects used a greater angular range (*p* < 0.001), and taller subjects used a greater angular range (*p* = 0.019). Age was the only covariate showing a significant effect on maximum angular velocity (*p* = 0.004) since angular velocity decreased with age.

### 3.3. Extension Phase

The duration of the extension phase tended to be longer for lower seat heights (*p* = 0.062) and was significantly longer for lower HGS (*p* = 0.003). The angular range was larger for lower seat heights (*p* = 0.002) and for lower HGS (*p* < 0.001) in the presence of a significant interaction effect of seat height and HGS (*p* < 0.001). Maximum angular velocity was higher for lower seat height (*p* < 0.001) and for lower HGS (*p* < 0.001). In addition, there was a significant seat height*HGS interaction (*p* = 0.002). Older subjects showed a lower angular velocity (*p* = 0.026). Vertical velocity was higher for lower seat heights (*p* < 0.001) and for higher HGS (*p* = 0.011). Furthermore, vertical velocity was higher for taller subjects (*p* = 0.048), lower for older ages (*p* = 0.025), and higher for males (*p* < 0.005).

**Figure 3:**
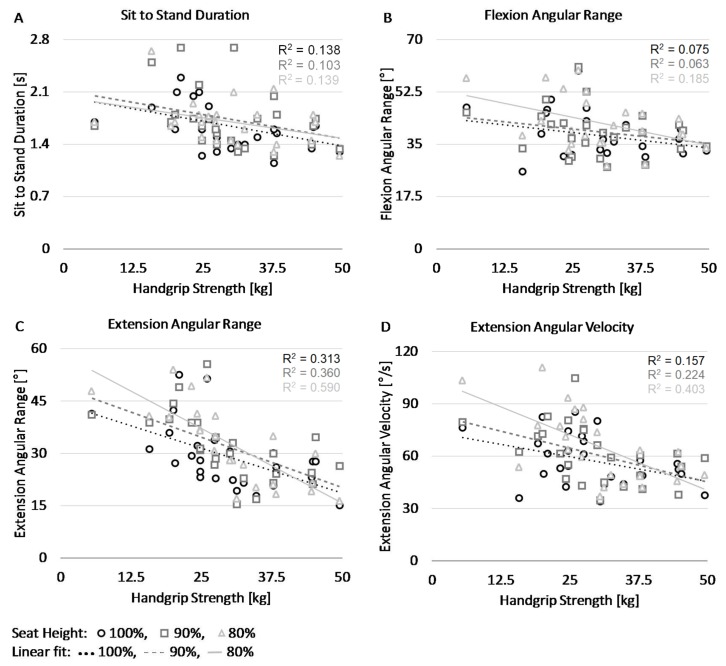
Scatter plots display values of the four STS parameters and handgrip strength at three different seat heights. Panel (**A**) shows the total duration of the SiSt phase. Panel (**B**) shows the angular range during the STS flexion phase. Panel (**C**) shows the angular range during the STS extension phase. Panel (**D**) shows the maximal angular velocity during the STS extension phase.

The most important outcomes are visualized in the scatterplots depicted in Figure 3. The four panels show the relation between handgrip strength (horizontal axes) and the STS duration, the flexion angular range, the extension angular range, and the extension angular velocity, respectively. As is evident from panel A, stronger subjects (i.e., with greater handgrip strength) performed the STS faster. Panel B shows that stronger subjects had a smaller flexion angular range while panel C shows that the weaker subjects had a much larger extension angular range, which increased with lower seat heights. Panel D shows a comparable effect for the maximum angular velocity during extension.

## 4. Discussion

The results showed that older adults with lower handgrip strength stand up with greater flexion of the trunk than older adults with higher handgrip strength. The weaker subjects also showed greater trunk extension with a higher angular velocity after seat-off, indicating a more dynamic use of the trunk. The hypothesis that older adults with lower muscle strength stand up with more dynamic use of the trunk was, therefore, confirmed. This finding is consistent with the results of Hughes et al. showing that functionally impaired elderly rising from chairs below knee height significantly increased peak hip flexion velocity and the time to rise when compared to rising from a knee height chair. In addition, they significantly decreased their mean center of mass/base of support (COM/BOS) separation at lift-off [16] in line with the suggestion that this strategy may facilitate balance control [13].

The single module instrumentation can be easily attached over undergarments beneath outer clothes in a manner that is unobtrusive to the subject. The participant’s awareness of being assessed is low because the instrumentation is not visible for the participant. Data collection is fast and, with the remote control, the test leader can stay in close contact with the participant. Moreover, the online connection of the remote control makes it possible for a single test leader to simultaneously collect data and watch over the participant since it is no longer necessary to read out the stopwatch and write down the times. The automated analysis of the data provides detailed insight into the quality of the movements.

Physical performance tests that include the STS such as the Timed Up and Go [35] and the repeated STS as a sub-test of the Short Physical Performance Battery [36] measure the duration of the total test and provide no information about the STS sub-durations and kinematics. The duration of the static periods (standing and sitting) during the 5× repeated STS represents, at least for some older adults, a great part of the total duration [27]. The total STS duration as measured with a stopwatch provides incomplete information about the dynamic phases of the STS, which are particularly relevant. The present findings demonstrate that kinematic parameters of standing up might be useful for clinical practice. Trunk range of motion, maximum angular velocity, and vertical velocity might be used to identify STS strategies in clinical practice and to evaluate interventions.

Chair height has a major influence on the ability to successfully stand up from a sitting position. A previous study reported that older adults found that, in general, the use of hands during STS transfer from different bed heights made the performance less challenging. All participants reported that the lowest position was the most challenging position to perform the task [37].

Using backwards elimination from all covariates, HGS was proven to be the strongest predictor of the durations as well as the kinematic outcomes. Older adults with lower handgrip strength, therefore, seemed to resort to a different STS strategy, which involved a more dynamic use of the trunk. The present and previous studies have shown that muscle strength is likely an important determinant of successfully standing up from a sitting position [10,12,38,39]. However, besides strength training, the training of STS transfer skills may well form a critical element in interventions aimed at preventing loss of mobility in older adults and patients [40]. Improvement of the technical performance of the STS might be more sustainable than strength training. Analysis of STS strategy using body fixed sensors in combination with easy-to-use strength measures will help to objectively measure changes over time and better understand how older adults could improve their STS ability.

Several determinants like chair seat height and lower limb strength have a major influence on the ability to successfully stand up from a sitting position. Increasing lower limb strength is a possible solution. Seat height may need to be more closely scrutinized in areas frequented by frail elderly people. Augmentation of seat height by small increments facilitates chair rise performance. In the present study, we manipulated seat height. Standing up from a lower seat height commonly occurs in daily life such as when standing up from a sofa. However, the seating area of a sofa is longer and it includes a backward slope and a compliant seat [41]. Therefore, standing up from a sofa might be even more difficult.

Adaptations of STS behavior have also been described in patients with other health conditions. Patients with knee osteoarthritis primarily shifted their COM closer to the base of support provided by the feet in an attempt to achieve stability at and after seat-off [42]. Additionally, individuals with Parkinson’s disease have difficulty rising from a chair as a result of reduced muscle strength, particularly at the hip amongst other factors [43]. In patients with a stroke, longer total time as well as changes at the initial phase and at the end of hip and knee extension phase were observed. The maximal hip flexion was lower during the rising phase from a seated position on the affected side in the stroke group [44]. Patients with end stage renal disease undergoing hemodialysis have high morbidity and mortality due to multiple causes. The results indicated that patients were slower to get out of the chair, which was measured by trunk flexion angular accelerations, time to peak trunk flexion, and overall STW completion time following the dialysis therapy session [45]. The present results add to these findings by showing that healthy older adults with reduced muscle strength also show significant adaptations in STS behavior, which is characterized by a more dynamic use of the trunk.

A limitation of the present study involves not measuring lower extremity strength and instead using HGS as a proxy for global muscle strength even though lower extremity muscle power was proven no better than knee extension torque or handgrip strength in the early identification of poor mobility [46]. Nevertheless, the change to a more dynamic STS strategy should be tested in a study protocol including lower leg strength measurements. To date, only a few studies have demonstrated the added value of using the instrumented STS in interventions [47,48]. The cross-sectional design of the present study precluded an analysis of how the STS strategy changes over time as muscle strength changes. It would be useful to examine whether older adults do revert to another STS strategy after an intervention is aimed at increasing lower leg strength. Longitudinal studies are needed to shed light on this issue. Finally, in the present study, using the arms during the STS procedure was prohibited by the protocol. The reason for this limitation was due to a focus specifically on the role of muscle strength and we, therefore, wished to eliminate the use of arm support or movement to compensate for lower leg strength. Future research could focus on how arm use might affect the STS dynamics.

## 5. Conclusions

Older adults with lower handgrip strength employed a different strategy to stand up from a sitting position than older adults with higher handgrip strength, which was characterized by more trunk flexion and a more dynamic use of the trunk during the extension phase. Changing to another strategy seems to be an adaptation of older adults to their reduced muscle strength. Measures of trunk kinematics were more sensitive to muscle strength than durations of STS phases.

## Figures and Tables

**Table 1:** Descriptive statistics of study population.

n	27
percentage male (n)	55.6% (15)
Age (years)	70.0 (67–83)
Height (cm)	173.4 ± 7.4
Weight (kg)	77.1 ± 13.2
BMI (kg/m^2^)	25.6 ± 3.8
Hand Grip strength (kg)	29.2 ± 10.1

Note: Data represents the mean ± SD or median (Q1–Q3).

**Table 2:** Durations (s), angular range (φ in °), angular velocity (ω_max_ in °/s) and vertical velocity (v_max_ in m/s) during flexion and extension of the Sit-to-Stand movement for the three seat heights. Model effects of Generalized Estimating Equations are displayed for the condition factor Seat Height and for the included covariates.

						Model Effects (*p*-Value) of GEE					
		Seat Height				Covariates					
		100%	90%	80%	Seat Height	HGS	Seat Height*HGS	Body Height	Body Weight	Age	Gender
**Sit-to-Stand**											
	Duration	1.60 (1.40–1.90)	1.65 (1.43–1.80)	1.70 (1.45–1.80)	0.097	0.015					
**Flexion phase**											
	Duration	0.76 (0.72–0.86)	0.81 (0.73–0.83)	0.82 (0.77–0.95)	0.071						
	Angular range	38.08 ± 7.58	39.28 ± 7.92	42.13 ± 8.96	0.003	<0.001		0.019			
	Angular velocity	113.18 (96.69–151.89)	117.10 (102.44–143.33)	119.31 (98.69–165.46)	0.201					0.004	
**Extension phase**											
	Duration	0.84 (0.69–1.03)	0.91 (0.71–1.03)	0.87 (0.74–0.96)	0.062	0.003					
	Angular range	29.40 ± 9.26	32.00 ± 9.82	32.83 ± 11.49	0.002	<0.001	0.001				
	Angular velocity	57.47 ± 14.47	61.04 ± 16.83	65.92 ± 20.64	<0.001	<0.001	0.002			0.026	
	Vertical velocity	0.59 ± 0.15	0.62 ± 0.18	0.67 ± 0.15	<0.001	0.011		0.048		0.025	0.005

Notes: Data represents the mean ± SD or median (Q1–Q3). An empty cell means that the covariate was eliminated prior to the last GEE iteration and hence no *p*-value was calculated.
